# Dataset on reproductive traits of Scandinavian alpine plants

**DOI:** 10.1016/j.dib.2019.104149

**Published:** 2019-06-12

**Authors:** Hans Henrik Bruun

**Affiliations:** University of Copenhagen, Department of Biology, Universitetsparken 15, 2100 Copenhagen, Denmark

**Keywords:** Plant reproductive biology, Alpine environment, Seed mass, Seed number

## Abstract

Data on reproductive traits of alpine plants in the central Scandes Mountains (Sweden) are given, namely seed mass, seed number per plant and seed number per unit area of vegetation. Data were obtained 1) by counting reproductive units (whole plants, flower heads, capsules, berries, as applicable) per meter squared in seven distinct vegetation types, 2) by counting the number of seeds per reproductive unit in the lab, and 3) by weighing seeds (discriminating between dispersule and germinule wherever relevant).

**Table:** Specifications table

Subject area	*Biology*
More specific subject area	*Plant reproductive ecology*
Type of data	*Tables*
How data was acquired	*Field data collection and lab measurement (Mettler MT5 balance)*
Data format	*Raw*
Experimental factors	*Alpine vegetation zones*
Experimental features	*Observational data on seed mass, seed number per plant and/or per unit area*
Data source location	*Sweden, Jämtland county, Helags area (62°55′N; 12°35′E)*
Data accessibility	*The data are available within this article*

**Table:** 

**Value of the data** • These original data were collected in 2002 in the Helags mountain area, Jämtland County, Sweden. • The data concern key plant functional traits related to sexual reproduction, namely offspring size, fecundity per mother plant and reproductive output per unit area. • The data may be used within the expanding field of trait-based community ecology, in which many published studies are bound to rely on standard values for species, i.e. previously collected data retrieved from databases and original publications. • Primary research in plant community ecology in the Arctic-Alpine realm of Europe and the wider Holarctic will probably find the data useful, not the least because, for many of the species here included, no data on seed mass or reproductive output have been published before. • The data may be used to improve insights gained from studies of seed bank formation and seed dispersal, in which own empirical data on seed mass and seed output per unit area are usually not available. Moreover, when included in a global trait database, the data may prove useful in macroecological analyses.

## 1 Data

Data are organized in three tables: 1) Site properties, 2) Seed mass data for 85 plant species, for many species measured with and without attached structures, such as pappus, and 3) Seed number per reproductive unit (whole ramet, flower head, capsule, berry etc.) and number of reproductive units per meter squared for a total of 105 species in seven vegetation types, each represented by 10 random sample plots. Seed number per ramet is given for 79 species, excluding cushion and mat forming species, for which ramets either are irrelevant or cannot be reasonably delimited.

## 2 Experimental design, materials, and methods

### 2.1 Geography

The Helags area (62°55′N; 12°35′E) in southern Jämtland County, Sweden, is situated in the central part of the Scandes Mountains. The highest peak locally, Helagsfjället, reaches an altitude of 1796 m a.s.l., and has Sweden's southernmost glacier. The climate is comparatively continental, with a January mean between −7 and −8 °C, a July mean of +8–10 °C, and a mean annual precipitation of 700–1200 mm. The bedrock is predominantly gneiss, but veins of calcareous schists occur frequently. The whole area is extensively grazed by semi-domestic reindeer.

### 2.2 Data on seed productivity and seed mass

A large-scale transect of c. 3 × 13 km was placed on the eastern macroslope of the Helags massive, from uppermost middle alpine zone and eastwards down to subalpine areas in the Ljungdalen valley. The seven most important vegetation types in terms of area were selected for sampling (Table 1): middle alpine grass heath, low alpine dwarf-shrub heaths of three types (poor, mesic and dry rich, mainly differing snow cover in winter and soil moisture in summer), low alpine moderate snowbed (short-herb meadow), and subalpine birch forest of two types (meadow type and heath type, differing in soil moisture and pH). These vegetation types collectively covered a total of 89% of the vegetated area of the transect (Andersson 2000). The excluded vegetation types were in general very wet or otherwise difficult to access (e.g. willow scrub). Fieldwork took place at the end of August 2002. For each vegetation type a representative area of suitable size was selected. Within each area 10 plots of 1 × 1 m were placed along a small-scale transect laid out in order to maximally cover the internal variation in microtopography (e.g., in the case of poor dwarf-shrub heath, which occurs on ridges with little or no snow cover during winter, the transect started at the most barren ridge-top and went downwards to parts with deeper winter snow-cover). Within each sample plot, the number of reproductive units of each species was counted. Reproductive units were ramets or individuals in most cases (42 and 15 species, respectively). For tussock-forming species, both the number of reproductive tussocks and the total number of reproductive shoots were counted. For most Asteraceae and other species with distinct flower heads, both the number of reproductive individuals/ramets and the number of flower heads were counted (11 species), and likewise for 3 species with individual flowers/fruits (e.g., *Viola biflora*). In cushion and mat forming plants (10 species), the reproductive unit counted were berries (e.g., *Vaccinium* spp.), capsules (e.g., *Loiseleuria procumbens*), catkins (*Betula nana* and *Salix herbacea*) or individual flowers (*Dryas octopetala*). All phenological stages of reproductive structures were included in counting. It was not possible to estimate the seed productivity (number of seeds per ramet or individual) for cushion and mat forming species (see Table 2 and 3).

**Table 1:** Data on the seven vegetation types sampled for seed yield: geographical coordinates and altitude of the sampled site (10 plots of 1 m^2^), sampling date, and the landscape-scale percentage cover (in the large-scale transect; lakes and barren block-fields excluded from the total) according to a vegetation map of the area [2].

Vegetation type	Site name	Long	Lat	Altitude (m a.s.l.)	Date sampled	Corresponding vegetation type on the map of Andersson (2000)	Percent landcover per vegetation type on the wider landscape scale
Subalpine birch forest of meadow type	1. Sweden, Jämtlands län, Ljungdalen, Postvallen	12.716461	62.899	760	28-08-2002	Birch forest of meadow type	10.3
Subalpine birch forest of heath type	2. Sweden, Jämtlands län, Ljungdalen, Kläppen	12.708967	62.897	820	28-08-2002	Moss-rich birch forest	8.7
Low alpine poor heath	3. Sweden, Jämtlands län, Ljungdalen, V.f. Kesusjön	12.606989	62.908	910	26-08-2002	Poor heath	7.0
Low alpine mesic heath	4. Sweden, Jämtlands län, Ö Helagsskaftet	12.578594	62.909	950	27-08-2002	Mesic heath	20.4
Low alpine moderate snowbed	5. Sweden, Jämtlands län, Ö Helagsskaftet	12.575511	62.910	970	26-08-2002	Short-herb meadow	11.2
Low alpine dry rich heath	6. Sweden, Jämtlands län, Ö Helagsskaftet	12.547966	62.910	1010	27-08-2002	Dry heath	27.0
Middle alpine grass heath	7. Sweden, Jämtlands län, Helags	12.482430	62.911	1280	27-08-2002	Grass heath	4.2

**Table 2:** Data on seed mass. Seed collections sites are numbered as in Table 1, except site 8, Sweden, Jämtlands län, west of Torkilstöten, site 9, Sweden, Jämtlands län, Sörvatnet, site 10, Sweden, Jämtlands län, Hamrafjället, and site 11, Sweden, Dalarna län, Idre socken, Strömmen. Figures in column 3 “No coll.” indicates the Number of individuals or ramets or reproductive units collected.

Species name	Seed collection site	No coll.	Reproductive unit	Dispersule/germinule	Dispersule/germinule explanation	Seed mass average (mg)	Seed mass SD	Number of seeds weighed
*Aconitum lycoctunum*	site 1	7	ramet	d; g	seed	3.625	0.798	10
*Alchemilla alpina*	site 5	10	ramet	d	nutlet + calyx	1.002	0.209	10
*Alchemilla alpina*	site 5	10	ramet	g	nutlet	0.479	0.168	10
*Angelica archangelica* ssp. *Archangelica*	site 11	5	ramet	d; g	schizachene	3.597	0.811	10
*Antennaria alpina*	site 7	10	ramet	d	achene + pappus	0.140	0.02	10
*Antennaria alpina*	site 7	10	ramet	g	achene	0.108	0.016	10
*Anthoxanthum odoratum*	site 5	15	ramet	d	caryopsis + glumes	0.615	0.07	10
*Anthoxanthum odoratum*	site 5	15	ramet	g	caryopsis	0.405	0.064	10
*Arctostaphylos alpinus*	site 8	10	berry/catkin	g	seed	2.404	0.605	10
*Bartsia alpina*	site 3	4	ramet	d; g	seed	0.246	0.081	10
*Betula nana*	site 3	15	berry/catkin	d; g	seed	0.267	0.089	10
*Carex atrata*	site 5	6	ramet	d	nutlet + utricle	0.958	0.142	10
*Carex atrata*	site 5	6	ramet	g	nutlet	0.805	0.135	10
*Carex atrofusca*	site 6	10	ramet	d	nutlet + utricle	0.405	0.065	10
*Carex atrofusca*	site 6	10	ramet	g	nutlet	0.302	0.054	10
*Carex bigelowii*	site 3	7	ramet	d	nutlet + utricle	0.695	0.273	10
*Carex bigelowii*	site 3		ramet	g	nutlet	0.581	0.24	10
*Carex capillaris*	site 6	11	ramet	d	nutlet + utricle	0.645	0.042	10
*Carex capillaris*	site 6	11	ramet	g	nutlet	0.466	0.034	10
*Carex lachenalii*	site 5	2	ramet	d	nutlet + utricle	0.424	0.042	10
*Carex lachenalii*	site 5	2	ramet	g	nutlet	0.334	0.035	10
*Carex norvegica* ssp. *Inferalpina*	site 3	2	ramet	d	nutlet + utricle	0.490	0.064	10
*Carex norvegica* ssp. *Inferalpina*	site 3	2	ramet	g	nutlet	0.369	0.039	10
*Carex saxatilis*	site 5	10	ramet	d	nutlet + utricle	0.656	0.09	10
*Carex saxatilis*	site 5	10	ramet	g	nutlet	0.495	0.075	10
*Carex vaginata*	site 9	8	ramet	d	nutlet + utricle	3.675	0.367	10
*Carex vaginata*	site 9	8	ramet	g	nutlet	2.137	0.166	10
*Cassiope hypnoides*	site 5	16	capsule	d; g	seed	0.013	0.001	10
*Cerastium cerastoides*	site 5	8	ramet	d; g	seed	0.202	0.029	10
*Cornus suecica*	site 2	10	berry/catkin	n	two-seeded stone	8.272	1.93	10
*Crepis paludosa*	site 1	10	ramet	d	achene + pappus	0.833	0.107	5
*Crepis paludosa*	site 1	10	ramet	g	achene	0.762	0.098	5
*Deschampsia cespitosa*	site 11	10	ramet	d	caryopsis + glumes	0.202	NA	1
*Deschampsia cespitosa*	site 11	10	ramet	g	caryopsis	0.117	NA	1
*Diapensia lapponica*	site 3	1	ramet	d; g	seed	0.051	0.004	10
*Dryas octopetala*	site 6	10	fertile shoot	d	nutlet with style	0.665	0.152	10
*Dryas octopetala*	site 6	10	fertile shoot	g	nutlet excl style	0.364	0.123	10
*Empetrum nigrum* ssp. *Hermaphroditum*	site 3	10	berry/catkin	g	seed	1.054	0.346	10
*Epilobium anagallidifolium*	site 5	10	ramet	d	achene with hairs	0.061	0.007	10
*Epilobium anagallidifolium*	site 5	10	ramet	g	achene	0.050	0.006	10
*Eriophorum angustifolium*	site 5	10	ramet	d	achene with hairs	1.161	0.178	10
*Eriophorum angustifolium*	site 5	10	ramet	g	achene	0.441	0.062	10
*Euphrasia frigida*	site 7	30	ramet	d; g	seed	0.169	0.048	10
*Euphrasia stricta*	site 5	7	ramet	d; g	seed	0.226	0.088	10
*Festuca ovina*	site 3	18	fertile shoot	d	caryopsis + glumes	0.468	0.1	10
*Festuca ovina*	site 3	18	fertile shoot	g	caryopsis	0.350	0.101	10
*Galium boreale*	site 1	10	ramet	d; g	schizachene	0.746	0.178	10
*Gentiana nivalis*	site 4	4	ramet	d; g	seed	0.046	0.007	10
*Hieracium alpinum*	site 6	1	ramet	d	achene + pappus	1.014	NA	1
*Hieracium alpinum*	site 6	1	ramet	g	achene	0.963	NA	1
*Juncus alpinus* ssp. *Alpestris*	site 3	5	ramet	d; g	seed	0.029	0.003	10
*Juncus biglumis*	site 5	12	ramet	d; g	seed	0.044	0.006	10
*Juncus castaneus*	site 3	2	ramet	d; g	seed	0.069	0.011	10
*Juncus trifidus*	site 3	30	capsule	d; g	seed	0.122	0.025	10
*Juncus triglumis*	site 5	1	ramet	d; g	seed	0.085	0.125	10
*Juniperus communis*	site 3	9	berry/catkin	g	seed	5.513	1.585	10
*Kobresia simpliciuscula*	site 6	10	ramet	d	nutlet + perigynium	0.571	0.089	10
*Kobresia simpliciuscula*	site 6	10	ramet	g	nutlet	0.521	0.096	10
*Koenigia islandica*	site 5	30	ramet	d; g	nutlet	0.321	0.073	10
*Linnaea borealis*	site 2	4	ramet	g	seed	1.552	NA	1
*Linnaea borealis*	site 2	4	ramet	d	fruit	2.403	0.395	6
*Loiseleuria procumbens*	site 3	30	capsule	d; g	seed	0.018	0.003	10
*Luzula arcuata*	site 7	20	ramet	d; g	seed	0.215	0.045	10
*Luzula spicata*	site 7	11	ramet	d; g	seed	0.405	0.055	10
*Melampyrum pratense*	site 4	10	ramet	d	seed with elaiosome	7.463	2.484	10
*Omalotheca norvegica*	site 1	2	ramet	d	achene + pappus	0.092	0.008	10
*Omalotheca norvegica*	site 3	1	ramet	d	achene + pappus	0.109	0.007	10
*Omalotheca norvegica*	site 1	2	ramet	g	achene	0.080	0.008	10
*Omalotheca norvegica*	site 3	1	ramet	g	achene	0.095	0.002	10
*Omalotheca supina*	site 5	10	ramet	d	achene + pappus	0.108	0.018	10
*Omalotheca supina*	site 5	10	ramet	g	achene	0.090	0.011	10
*Oxyria digyna*	site 3	7	ramet	d	nutlet + tepals	1.133	0.144	10
*Oxyria digyna*	site 3	7	ramet	g	nutlet	0.693	0.093	10
*Parnassia palustris*	site 11	1	ramet	d; g	seed	0.080	0.011	10
*Pedicularis lapponica*	site 7	2	ramet	d; g	seed	0.490	NA	1
*Pedicularis oederi*	site 3	3	ramet	d; g	seed	0.777	0.213	10
*Pedicularis sceptrum-carolinum*	site 4	2	ramet	d; g	seed	0.558	0.101	10
*Phleum alpinum*	site 3	5	ramet	d	caryopsis + glumes	0.533	0.069	10
*Phleum alpinum*	site 3	10	ramet	g	caryopsis	0.363	0.051	10
*Phyllodoce caerulea*	site 4	10	ramet	d; g	seed	0.012	0.001	10
*Pinguicula vulgaris*	site 6	4	ramet	d; g	seed	0.020	0.003	10
*Poa supina*	site 6	8	ramet	d	caryopsis + glumes	0.512	0.156	10
*Poa supina*	site 6	8	ramet	g	caryopsis	0.362	0.125	10
*Polygonum viviparum*	site 5	10	ramet	d; g	bulbil	1.559	0.598	10
*Potentilla crantzii*	site 3	1	ramet	d; g	nutlet	0.440	0.249	10
*Potentilla erecta*	site 1	2	ramet	d; g	nutlet	0.770	0.089	7
*Primula stricta*	site 6	3	ramet	d; g	seed	0.059	0.011	10
*Ranunculus acris* ssp. *Acris*	site 6	3	ramet	d; g	nutlet	1.481	0.268	10
*Ranunculus glacialis*	site 7	1	ramet	d; g	nutlet	0.378	0.115	10
*Ranunculus platanifolius*	site 10	5	ramet	d; g	nutlet	7.900	0.555	10
*Rhinanthus groenlandicus*	site 1	10	ramet	d; g	seed	1.974	0.337	10
*Rumex acetosa*	site 5	10	ramet	d	nutlet + tepals	1.286	0.299	10
*Rumex acetosa*	site 5	10	ramet	g	nutlet	0.883	0.226	10
*Salix herbacea*	site 5	30	capsule	d	achene + pappus	0.145	0.057	10
*Salix herbacea*	site 5	30	capsule	g	achene	0.086	0.028	10
*Saussurea alpina*	site 7	10	ramet	d	achene + pappus	2.067	0.446	10
*Saussurea alpina*	site 7	10	ramet	g	achene	1.541	0.363	10
*Saxifraga aizoides*	site 3	64	capsule	d; g	seed	0.058	0.01	10
*Saxifraga oppositifolia*	site 6	15	capsule	d; g	seed	0.088	0.01	10
*Saxifraga stellaris*	site 5	28	capsule	d; g	seed	0.029	0.006	10
*Sibbaldia procumbens*	site 5	25	fertile shoot	d; g	nutlet	0.552	0.053	10
*Silene acaulis*	site 7	15	capsule	d; g	seed	0.481	0.033	10
*Solidago virgaurea*	site 4	4	ramet	d	achene + pappus	0.740	0.115	10
*Solidago virgaurea*	site 4	4	ramet	g	achene	0.675	0.117	10
*Stellaria borealis*	site 11	5	ramet	d; g	seed	0.176	0.015	10
*Thalictrum alpinum*	site 3	5	ramet	d; g	nutlet	0.857	0.348	10
*Tofieldia pusilla*	site 6	14	ramet	d; g	seed	0.043	0.008	10
*Trientalis europaea*	site 2	1	ramet	d; g	seed	0.674	0.158	9
*Trientalis europaea*	site 4	4	ramet	d; g	seed	0.559	0.082	10
*Trifolium pratense*	site 1	7	flower head	g	seed	1.252	0.123	10
*Trifolium pratense*	site 1	7	flower head	d	calyx + pod + seed	2.334	0.141	10
*Trisetum spicatum*	site 7	1	ramet	d	caryopsis + glumes	0.381	0.106	3
*Trisetum spicatum*	site 7	1	ramet	g	caryopsis	0.225	0.075	3
*Vaccinium myrtillus*	site 4	10	berry/catkin	g	seed	0.201	0.029	10
*Vaccinium uliginosum*	site 9	7	berry/catkin	g	seed	0.222	0.06	10
*Vaccinium vitis-idaea*	site 4	9	berry/catkin	g	seed	0.236	0.061	10
*Valeriana sambucifolia*	site 1	5	ramet	d	achene + pappus	1.077	0.068	10
*Valeriana sambucifolia*	site 1	5	ramet	g	achene	0.925	0.06	10
*Veronica alpina*	site 5	6	ramet	d; g	seed	0.051	0.009	10
*Viola biflora*	site 4	4	ramet	d; g	seed with elaiosome	0.740	0.116	10

**Table 3:** Data on seed number per ramet and per unit area. For each vegetation type sampled, the number of sample plots, in which each species was observed is given in column 5–11 (NA means that the species was present, but that sexual reproduction was not observed). The total number of reproductive units per species per site (vegetation type) is given in columns 12–19. The estimated number of seeds produced per meter squared is obtained by multiplying the latter figure with the mean number of seeds per reproductive unit (column 4).

Species name	Number of plots per site in which the species was found as generative										Number of reproductive units per species per site						
	Reproductive unit sampled	Seed number per reproductive unit	Seed number per plant (ramet)	Subalpine birch forest of meadow type	Subalpine birch forest of heath type	Low alpine poor heath	Low alpine mesic heath	Low alpine moderate snowbed	Low alpine dry rich heath	Middle alpine grass heath	Subalpine birch forest of meadow type	Subalpine birch forest of heath type	Low alpine poor heath	Low alpine mesic heath	Low alpine moderate snowbed	Low alpine dry rich heath	Middle alpine grass heath
*Aconitum lycoctunum*	fertile shoot	315.9	315.9	2	0	0	0	0	0	0	7	0	0	0	0	0	0
*Agrostis capillaris*	fertile shoot	58.8	58.8	9	1	0	0	0	0	0	60	5	0	0	0	0	0
*Agrostis mertensii*	fertile shoot	34.5	34.5	0	0	0	0	5	1	3	0	0	0	0	18	1	29
*Alchemilla alpina*	fertile shoot	45.8	45.8	0	0	0	0	1	0	1	0	0	0	0	280	0	3
*Alchemilla* sp.	fertile shoot	51.9	51.9	1	0	0	0	0	0	0	1	0	0	0	0	0	0
*Andromeda polifolia*	NA	NA	NA	0	0	0	0	0	NA	0	0	0	0	0	0	0	0
*Angelica sylvestris*	NA	NA	NA	NA	0	0	0	0	0	0	0	0	0	0	0	0	0
*Antennaria alpina*	fertile shoot	8.0	8.0	0	0	0	0	0	2	1	0	0	0	0	0	7	7
*Anthoxanthum odoratum*	fertile shoot	15.6	15.6	6	1	0	0	4	3	0	11	1	0	0	0	15	0
*Arctostaphylos alpinus*	NA	NA	NA	0	0	NA	0	0	NA	0	0	0	0	0	0	0	0
*Bartsia alpina*	fertile shoot	352.1	352.1	1	0	0	0	0	NA	0	1	0	0	0	0	0	0
*Betula nana*	berry/catkin	39.2	NA	0	NA	8	5	0	2	0	0	0	72	143	0	18	0
*Carex atrata*	fertile shoot	65.5	65.5	0	0	0	0	0	1	0	0	0	0	0	0	1	0
*Carex atrofusca*	fertile shoot	24.5	24.5	0	0	0	0	0	1	0	0	0	0	0	0	1	0
*Carex bigelowii*	fertile shoot	4.4	4.4	0	0	6	0	8	8	9	0	0	9	0	0	61	180
*Carex capillaris*	fertile shoot	9.3	9.3	0	0	0	0	0	1	0	0	0	0	0	0	9	0
*Carex lachenalii*	fertile shoot	21.0	21.0	0	0	0	0	3	0	0	0	0	0	0	0	0	0
*Carex saxatilis*	fertile shoot	43.8	43.8	0	0	0	0	4	0	0	0	0	0	0	49	0	0
*Carex vaginata*	fertile shoot	5.0	5.0	1	0	0	0	0	1	0	1	0	0	0	0	2	0
*Cassiope hypnoides*	capsule	95.4	95.4	0	0	0	0	2	0	2	0	0	0	0	0	0	12
*Cerastium cerastoides*	capsule	23.8	35.7	0	0	0	0	2	0	0	0	0	0	0	3	0	0
*Cerastium fontanum* ssp. *Fontanum*	fertile shoot	219.2	219.2	1	0	0	0	0	0	0	1	0	0	0	0	0	0
*Cicerbita alpina*	fertile shoot	306.0	306.0	2	0	0	0	0	0	0	4	0	0	0	0	0	0
*Cirsium helenoides*	flower head	NA	NA	4	0	0	0	0	0	0	5	0	0	0	0	0	0
*Cornus suecica*	berry/catkin	1.0	NA	0	5	0	0	0	0	0	0	24	0	0	0	0	0
*Crepis paludosa*	flower head	9.5	17.1	10	0	0	0	0	0	0	108	0	0	0	0	0	0
*Deschampsia cespitosa*	fertile shoot	0.0	58.4	8	0	0	0	1	0	NA	27	0	0	0	0	0	0
*Deschampsia flexuosa*	fertile shoot	22.6	22.6	0	7	0	3	2	1	NA	0	37	0	3	0	3	0
*Diapensia lapponica*	capsule	62.6	62.6	0	0	1	0	0	0	1	0	0	6	0	0	0	3
*Dryas octopetala*	flower head	15.5	15.5	0	0	0	0	0	2	0	0	0	0	0	0	38	0
*Empetrum nigrum* ssp. *Hermaphroditum*	berry/catkin	7.4	NA	0	3	10	10	NA	7	NA	0	19	1130	236	0	350	0
*Epilobium anagallidifolium*	capsule	71.1	78.2	0	0	0	0	2	0	0	0	0	0	0	0	0	0
*Eriophorum angustifolium*	fertile shoot	29.8	29.8	0	0	0	0	3	0	0	0	0	0	0	0	0	0
*Euphrasia frigida*	ramet	10.2	10.2	0	0	0	0	0	4	3	0	0	0	0	0	39	93
*Euphrasia stricta*	ramet	18.5	18.5	2	0	1	0	1	0	0	21	0	25	0	0	0	0
*Festuca ovina*	fertile shoot	10.3	10.3	0	0	4	1	1	2	0	0	0	49	3	0	26	0
*Festuca vivipara*	NA	NA	NA	0	0	0	0	0	NA	NA	0	0	0	0	0	0	0
*Filipendula ulmaria*	fertile shoot	1246.4	1246.4	2	0	0	0	0	0	0	4	0	0	0	0	0	0
*Galium boreale*	fertile shoot	1.0	1.0	6	0	0	NA	0	0	0	44	0	0	0	0	0	0
*Geranium sylvaticum*	fertile shoot	9.9	9.9	9	0	0	0	0	0	0	65	0	0	0	0	0	0
*Geum rivale*	flower head	NA	NA	2	0	0	0	0	0	0	12	0	0	0	0	0	0
*Hieracium alpinum*	flower head	2.0	2.0	0	0	0	0	NA	1	NA	0	0	0	0	0	2	0
*Hieracium* sp.	NA	NA	NA	0	0	0	NA	0	0	0	0	0	0	0	0	0	0
*Juncus biglumis*	ramet	57.2	57.2	0	0	0	0	4	0	2	0	0	0	0	0	0	43
*Juncus trifidus*	fertile shoot	10.5	10.5	0	0	2	1	1	3	2	0	0	68	19	0	171	28
*Juncus triglumis*	fertile shoot	71.9	71.9	0	0	0	0	1	0	0	0	0	0	0	0	0	0
*Juniperus communis*	berry/catkin	2.4	70.1	1	2	0	NA	0	0	0	2	84	0	0	0	0	0
*Kobresia simpliciuscula*	fertile shoot	14.9	14.9	0	0	0	0	0	1	0	0	0	0	0	0	1	0
*Koenigia islandica*	ramet	1.3	1.3	0	0	0	0	1	0	0	0	0	0	0	0	0	0
*Linnaea borealis*	capsule	1.0	1.7	0	1	0	0	0	0	0	0	4	0	0	0	0	0
*Loiseleuria procumbens*	capsule	30.0	30.0	0	0	9	0	NA	3	0	0	0	449	0	0	133	0
*Luzula arcuata*	fertile shoot	14.4	14.4	0	0	0	0	0	0	3	0	0	0	0	0	0	47
*Luzula multiflora* ssp. *Frigida*	fertile shoot	NA	NA	0	1	0	0	0	0	0	0	2	0	0	0	0	0
*Luzula pilosa*	NA	NA	NA	NA	NA	0	0	0	0	0	0	0	0	0	0	0	0
*Luzula spicata*	fertile shoot	29.2	29.2	0	0	0	0	0	2	3	0	0	0	0	0	4	8
*Maianthemum bifolium*	NA	NA	NA	NA	NA	0	0	0	0	0	0	0	0	0	0	0	0
*Melampyrum pratense*	ramet	7.0	7.0	2	7	0	2	0	0	0	3	11	0	4	0	0	0
*Melica nutans*	fertile shoot	15.8	15.8	3	0	0	0	0	0	0	23	0	0	0	0	0	0
*Nardus stricta*	fertile shoot	12.3	12.3	0	3	0	0	7	2	0	0	17	0	0	0	12	0
*Omalotheca norvegica*	fertile shoot	1107.0	1107.0	1	1	0	0	0	0	0	2	1	0	0	0	0	0
*Omalotheca supina*	ramet	13.3	13.3	0	0	0	0	5	0	2	0	0	0	0	0	0	11
*Oxalis acetosella*	NA	NA	NA	NA	0	0	0	0	0	0	0	0	0	0	0	0	0
*Oxyria digyna*	fertile shoot	60.1	60.1	0	0	0	0	1	0	0	0	0	0	0	0	0	0
*Parnassia palustris*	ramet	89.0	89.0	1	0	0	0	0	0	0	1	0	0	0	0	0	0
*Pedicularis lapponica*	ramet	0.5	0.5	0	0	0	NA	0	0	1	0	0	0	0	0	0	2
*Pedicularis oederi*	NA	NA	NA	0	0	0	0	0	NA	0	0	0	0	0	0	0	0
*Phleum alpinum*	fertile shoot	79.4	79.4	1	0	0	0	1	0	0	1	0	0	0	0	0	0
*Phyllodoce caerulea*	fertile shoot	230.0	230.0	0	0	0	0	NA	5	NA	0	0	0	0	0	76	0
*Pinguicula vulgaris*	capsule	100.0	100.0	0	0	0	0	NA	1	0	0	0	0	0	0	5	0
*Poa alpigena*	fertile shoot	NA	NA	0	0	0	0	2	0	0	0	0	0	0	0	0	0
*Poa supina*	fertile shoot	36.5	36.5	0	0	0	0	0	1	0	0	0	0	0	0	7	0
*Polygonatum verticillatum*	NA	NA	NA	NA	0	0	0	0	0	0	0	0	0	0	0	0	0
*Polygonum viviparum*	ramet	17.9	17.9	3	0	0	0	4	6	9	31	0	0	0	0	36	59
*Potentilla crantzii*	flower head	4.5	49.5	0	0	0	0	0	NA	1	0	0	0	0	0	0	1
*Potentilla erecta*	fertile shoot	9.8	9.8	10	0	0	0	0	0	0	220	0	0	0	0	0	0
*Primula stricta*	ramet	205.2	205.2	0	0	0	0	0	1	0	0	0	0	0	0	1	0
*Ranunculus acris* ssp. *Acris*	flower head	7.7	7.7	6	0	0	0	0	2	0	14	0	0	0	0	5	0
*Ranunculus glacialis*	flower head	21.0	21.0	0	0	0	0	0	0	1	0	0	0	0	0	0	2
*Rhinanthus groenlandicus*	ramet	49.5	49.5	2	0	0	0	0	0	0	64	0	0	0	0	0	0
*Rhodiola rosea*	NA	NA	NA	0	0	0	0	0	0	NA	0	0	0	0	0	0	0
*Rubus chamaemorus*	NA	NA	NA	0	0	0	0	NA	0	0	0	0	0	0	0	0	0
*Rubus saxatilis*	berry/catkin	1.0	NA	1	0	0	0	0	0	0	1	0	0	0	0	0	0
*Rumex acetosa*	fertile shoot	62.4	62.4	6	3	0	0	2	0	0	17	12	0	0	0	0	0
*Sagina nivalis*	capsule	NA	NA	0	0	0	0	1	0	0	0	0	0	0	0	0	0
*Sagina saginoides*	capsule	NA	199.90	0	0	0	0	0	1	0	0	0	0	0	0	4	0
*Salix herbacea*	fertile shoot	23.9	23.9	0	0	NA	0	7	NA	6	0	0	0	0	0	0	129
*Saussurea alpina*	flower head	8.6	23.2	1	0	0	0	0	NA	2	6	0	0	0	0	0	4
*Saxifraga aizoides*	capsule	30.2	60.3	0	0	0	0	1	0	0	0	0	0	0	0	0	0
*Saxifraga oppositifolia*	capsule	17.7	17.7	0	0	0	0	0	2	0	0	0	0	0	0	14	0
*Saxifraga stellaris*	ramet	160.8	160.8	0	0	0	0	2	0	1	0	0	0	0	0	0	1
*Sibbaldia procumbens*	flower head	5.1	24.5	0	0	0	0	4	0	1	0	0	0	0	0	0	9
*Silene acaulis*	capsule	10.8	10.8	0	0	0	0	0	4	5	0	0	0	0	0	116	369
*Solidago virgaurea*	flower head	26.6	186.5	NA	4	0	NA	NA	1	0	0	84	0	0	0	7	0
*Taraxacum* sect. *Palustria*	NA	NA	NA	0	0	0	0	NA	0	0	0	0	0	0	0	0	0
*Thalictrum alpinum*	ramet	2.6	2.6	NA	0	0	0	NA	3	1	0	0	0	0	0	15	1
*Tofieldia pusilla*	ramet	145.1	145.1	0	0	0	0	0	2	0	0	0	0	0	0	5	0
*Trientalis europaea*	berry/catkin	8.2	8.8	NA	1	0	1	0	0	0	0	1	0	3	0	0	0
*Trifolium pratense*	flower head	42.7	85.4	1	0	0	0	0	0	0	2	0	0	0	0	0	0
*Trisetum spicatum*	fertile shoot	8.4	8.4	0	0	0	0	0	0	2	0	0	0	0	0	0	5
*Vaccinium myrtillus*	berry/catkin	11.7	NA	1	10	0	10	0	4	NA	2	365	0	537	0	12	0
*Vaccinium uliginosum*	berry/catkin	20.1	NA	0	NA	2	4	0	NA	0	0	0	13	31	0	0	0
*Vaccinium vitis-idaea*	berry/catkin	9.2	NA	1	5	NA	4	0	1	NA	3	51	0	31	0	6	0
*Veronica alpina*	ramet	75.2	75.2	0	0	0	0	2	1	2	0	0	0	0	0	3	5
*Vicia cracca*	NA	NA	NA	NA	0	0	0	0	0	0	0	0	0	0	0	0	0
*Viola biflora∖*	capsule	18.3	21.9	NA	0	0	1	1	NA	NA	0	0	0	2	0	0	0

At each site, ten reproductive units per (newly encountered) species were collected. This means that data on seed mass and seed number per reproductive unit were not gathered for all species at all sites, but at least once for the whole study area. If ripe fruits were not to be found in the sample plots, they were looked for in the vicinity or, in when necessary, in the greater region.

For all collections, the number of fully developed ripe seeds per reproductive unit was counted under a dissection microscope in the laboratory. The number of reproductive units per m^2^ may be multiplied by the number of seeds per unit in order to give the seed yield (number of seeds per m^2^). Seeds were stored in paper envelopes at room temperature and ambient moisture for two-four weeks, and subsequently 10 randomly selected seeds were weighed individually on a Mettler MT5 balance (nominal accuracy 0.001 mg). However, wherever relevant, separate measurements were made for dispersule (dispersal units leaving the mother plant, including various appendages, which may play a role in dispersal) and germinule (the after-dispersal structure, i.e. seed or small indehiscent fruit, such as achene and caryopsis) of the same species [1].
